# Overview of Glycometabolism of Lactic Acid Bacteria During Freeze-Drying: Changes, Influencing Factors, and Application Strategies

**DOI:** 10.3390/foods14050743

**Published:** 2025-02-22

**Authors:** Tchouli Noufeu, Yueqin Li, Ndeye Fatou Toure, Hui Yao, Xiaoqun Zeng, Qiwei Du, Daodong Pan

**Affiliations:** 1 State Key Laboratory for Quality and Safety of Agro-Products, Ningbo University, Ningbo 315211, China; 2 College of Food Science and Engineering, Ningbo University, Ningbo 315800, China; 3 Zhejiang-Malaysia Joint Research Laboratory for Agricultural Product Processing and Nutrition, Ningbo University, Ningbo 315800, China

**Keywords:** lactic acid bacteria, freeze-drying, glycometabolism, glycolytic, adenosine triphosphate, exopolysaccharides

## Abstract

Lactic acid bacteria (LAB) play a vital role in food fermentation and probiotics microeconomics. Freeze-drying (FD) is a commonly used method for preserving LAB powder to extend its shelf life. However, FD induces thermal, osmotic, and mechanical stresses that can impact the glycometabolism of LAB, which is the process of converting carbohydrates into energy. This review explores the effect of FD on glycometabolism, factors influencing glycometabolism, and feasible strategies in the FD process of LAB. During the three stages of FD, freezing, primary drying or sublimation, and second drying, the glycolytic activity of LAB is disrupted in the freezing stage; further, the function of glycolytic enzymes such as hexokinase, phosphofructokinase, and pyruvate kinase is hindered, and adenosine triphosphate (ATP) production drops significantly in the sublimation stage; these enzyme activities and ATP production nearly cease and exopolysaccharide (EPS) synthesis alters during the secondary drying stage. Factors such as strain variations, pretreatment techniques, growth medium components, FD parameters, and water activity influence these changes. To counteract the effects of FD on LAB glycometabolism, strategies like cryoprotectants, encapsulation, and genetic engineering can help preserve their glycometabolic activity. These methods protect LAB from harsh FD conditions, safeguarding glycolytic flux and enzymatic processes involved in carbohydrate metabolism. A deeper understanding of these glycometabolic changes is essential for optimizing FD processes and enhancing the use of LAB in food, medicine, and biotechnology, ultimately improving their performance upon rehydration.

## 1. Introduction

Lactic acid bacteria (LAB) are a group of microorganisms that play a crucial role in various food processes, including the fermentation of foods like yogurt, cheese, and sauerkraut to improve their flavor, texture, and shelf life [1]. In addition to their role in fermentation, LAB also function as probiotics, which are beneficial microorganisms for digestive improvement, immune modulation, and gastrointestinal disorder prevention when consumed in adequate amounts [2]. Another essential characteristic of LAB is its ability to produce lactic acid, a process that serves as a natural preservative, preventing the growth of harmful bacteria and extending the shelf life of the food [3]. The importance of LAB storage lies in preserving their viability, stability, and functionality, which are essential for their applications in the food and pharmaceutical industries [4].

Drying methods such as freeze-drying (FD) and spray-drying are frequently used to preserve probiotics and starter cultures [5]. FD, or lyophilization, effectively preserves LAB’s viability and metabolic activity by removing water from their cells. It is preferred for preserving probiotics because it helps maintain their viability, extends shelf life, improves stability, and makes transportation and storage easy, which results in a convenient, versatile product for manufacturers and consumers [6,7], allowing years of storage without loss of function [8]. While FD is often considered expensive, it offers cost advantages in specific contexts, such as minimal equipment needs at the laboratory scale [9,10]. There are three main steps in the process: (i) complete freezing, which involves solidifying the product through freezing at low temperatures and creating an ice crystal-based matrix enclosing the solute; (ii) sublimation or primary drying, which starts by raising the shelf temperature while applying a vacuum; and (iii) the desorption of leftover unfrozen or bonded water, also known as secondary drying [11]. During freeze-drying, LAB experiences various stresses, including thermal stress (from freezing and sublimation), osmotic stress (as water is removed, the concentration of solutes increases), starve stress, and mechanical stress (due to the formation of ice crystals and changes in physical structure) [12]. Stress responses can cause changes in the activity of enzymes involved in glycometabolic pathways (Table 1). These enzymes that facilitate carbohydrate synthesis and breakdown may be upregulated or downregulated in response to stress, affecting the overall balance of carbohydrate metabolism [13].

LAB relies on carbohydrate metabolism to survive and grow. This makes glycometabolism, the process by which cells transform carbohydrates into energy, crucial for the protection and survival of LAB during FD [21]. Adenosine triphosphate (ATP) produced by glycolysis is indeed crucial for various cellular processes, including protein synthesis, the maintenance of cellular structures, and the activation of repair mechanisms. ATP is required to fuel the repair of damage caused by oxidative stress, membrane damage, and protein misfolding that often occurs during FD [18,22]. Acidic metabolites, such as lactate, produced during glycometabolism can help regulate intracellular pH. Maintaining a stable pH is crucial for preserving enzymatic activity and metabolic processes in LAB, minimizing the harmful effects of environmental stress [23]. During lyophilization, maintaining intracellular pH stability is challenging in frozen or dry environments due to the lack of water [24]. However, the buffering capacity of the intracellular environment and pH-stabilizing agents can help maintain stability. Measuring pH after rehydration or using chemical indicators can provide insights, ensuring optimal enzymatic activity even under stress. The choice and application of cryoprotectants such as trehalose, a protective disaccharide, as well as the parameters of freezing and drying procedures, all contribute significantly to the success of lyophilization in helping LAB cells stay alive and maintain their glycometabolic activity [13]. This interplay of factors demonstrates how complex glycometabolism and lyophilization are in LAB.

Understanding changes in glycometabolism during FD is crucial not only for the preservation of LAB but also for developing improved lyophilization protocols for other biotechnological applications. This review presents the various stages of LAB FD, their advantages and disadvantages, and general information on glycometabolism in LAB. Additionally, we discuss the glycometabolism modifications of LAB induced by FD. Concurrently, we examine the factors influencing glycometabolism during the process and the strategies to mitigate the effects of lyophilization. This knowledge can help select and formulate appropriate preservation for probiotic LAB strains, enhancing their survival and functionality in various products. To compile this review, we performed an extensive literature search across several reputable databases, including PubMed, Google Scholar, and Scopus. We selected articles based on their relevance to the topic, methodological quality, and their contributions to advancing our understanding of LAB preservation and the glycometabolic changes that occur during FD.

## 2. Freeze-Drying of Lactic Acid Bacteria

**Figure 1:**
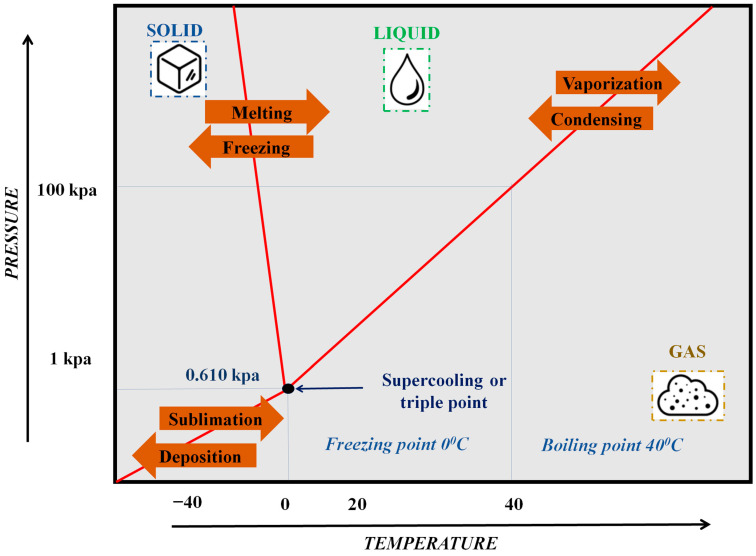
The process of freeze-drying LAB, which involes three stages that remove water by freezing it and placing it under specific vacuum pressures and temperatures.

FD is a dehydration process that preserves products by freezing them, reducing pressure, and sublimating the ice directly into vapor, bypassing the liquid phase [25]. This process involves three main stages: freezing, primary drying (sublimation), and secondary drying (desorption) (Figure 1). Initially, the product is frozen, concentrating the solutes into a frozen concentrate. During primary drying, the pressure is lowered and the temperature is increased to sublime the ice, forming a porous structure. Secondary drying removes the remaining water by desorption.

### 2.1. Freezing Stage

The freezing stage plays a pivotal role in preserving the viability and glycometabolic stability of LAB during FD, necessitating precise regulation of nucleation and phase transitions to minimize cellular damage [26]. The cooling rate is a key factor influencing ice crystal formation, slow cooling encourages the development of large ice crystals that can compromise cell membrane integrity, whereas rapid cooling facilitates the formation of smaller, more uniform crystals, mitigating mechanical stress [27]. To manage this process effectively, controlled nucleation techniques such as gas depressurization, ultrasound-assisted freezing, and ice seeding can be employed to standardize ice formation, thereby enhancing cellular protection [28,29]. Moreover, the freezing temperature itself plays a crucial role. Typically, temperatures are brought down to below −40 °C, ensuring that all the water within the bacterial suspension is frozen.

During the phase transition, water within bacterial cells transitions from a liquid to a solid state. Regulating this shift is essential to prevent intracellular ice formation, which can be fatal to the cells [27,30]. In certain conditions, intracellular water can solidify without crystallization through vitrification, a process facilitated by cryoprotectants such as trehalose, glycerol, and dimethyl sulfoxide (DMSO). These compounds penetrate the cell membrane, replace intracellular water, and induce the formation of a glass-like, amorphous structure that preserves cellular integrity [31]. Additionally, elevated solute concentrations increase viscosity, inhibit nucleation, and mitigate freeze-induced stress, further protecting LAB during lyophilization [32]. By fine-tuning cooling rates, nucleation strategies, and vitrification conditions, FD protocols can be optimized to enhance LAB survival and maintain functionality, ensuring their efficacy in industrial applications.

### 2.2. Primary Drying or Sublimation Stage

The sublimation stage, or first drying phase, of FD is a critical process during which LAB are exposed to stresses such as dehydration, oxidative stress, and changes in osmotic pressure, which can significantly affect the function of glycolytic enzymes, further limiting adenosine triphosphate (ATP) production [27]. This step that involves the direct transition of ice from a solid to a vapor state without passing through a liquid phase. This is achieved by maintaining the product at temperatures below its eutectic point and applying a vacuum to reduce the surrounding pressure. Initially, the ice within the bacterial culture sublimates as heat is transferred to the product [33]. This heat transfer is typically conducted through conduction, radiation, or convection, ensuring that the temperature remains low enough to avoid damaging the delicate bacteria and high enough to sustain the sublimation process. Controlling both pressure and temperature is vital during this stage to ensure efficient sublimation. The vacuum must be precisely regulated to maintain a pressure that favors sublimation, usually within the 0.1 to 1.0 mbar range. Concurrently, the shelf temperature is often controlled to provide the necessary latent heat for sublimation without causing thermal degradation [34].

Precise endpoint detection during sublimation is crucial in FD to prevent incomplete sublimation, which can compromise structural integrity and lead to bacterial deterioration. The endpoint can be measured using a measurement sensor, or an infrared camera for FD process control [35,36]. Jeyapradhap et al. proposed a sensor system, integrating a velocity sensor, an arduino nano, and a Bluetooth module, which are strategically placed between the product chamber and condenser, allowing real-time tracking of sublimation and water desorption for precise endpoint detection [35]. However, the most reliable method involves monitoring product temperature using thermocouples or resistance-based sensors embedded within the sample, as chamber temperature alone does not accurately reflect drying progress. Additionally, non-invasive analytical techniques such as tunable diode laser absorption spectroscopy (TDLAS), comparative pressure measurement, and mass spectrometry have been widely employed to determine the completion of primary drying by analyzing residual moisture levels and vapor concentration shifts [37]. Furthermore, techniques and monitoring tools such as the Pirani gauge, capacitive sensors, dew point monitoring, and pressure rise test (PRT) provide highly accurate endpoint determination, ensuring precision and process reliability. Two-chamber freeze-dryers with closable intermediate valves are essential for conducting PRT [38]. By incorporating cutting-edge monitoring technologies and optimizing vacuum pressure, heat transfer, and sample temperature, the primary drying phase can be fine-tuned to enhance LAB viability and produce a stable, high-quality freeze-dried product [39].

### 2.3. Secondary Drying or Desorption Stage

The third stage of FD, known as the secondary drying or desorption phase, plays a crucial role in the preservation and stability of LAB. Following primary drying, where ice sublimates, the second drying focuses on further reducing residual moisture levels to enhance product stability and shelf life [40]. This stage aims to achieve the desired moisture content critical for long-term storage and reconstitution of LAB cultures. The transition from primary to secondary drying in lyophilization demands the precise regulation of temperature and pressure to maintain product integrity. During this phase, chamber pressure is typically controlled within 13 to 27 Pa, while product temperature is gradually increased to a level above ambient but within the thermal tolerance of the formulation (20 °C to 40 °C). This controlled heating phase promotes the efficient removal of residual bound water through desorption, ensuring optimal product stability and extended shelf life. This phase is essential for preserving the viability and functionality of LAB, as excessive moisture can lead to microbial growth or the degradation of cellular components [41].

The estimation of the desorption rate is a common technique for monitoring secondary drying, which can be performed by PRT and tunable diode laser absorption spectroscopy (TDLAS), but their accuracy can be limited by low desorption rates. A correlation between pressure increases during PRT and residual moisture was proposed to determine the secondary drying endpoint. However, the low desorption rate at this stage reduces measurement accuracy, making it unsuitable for determining optimal secondary drying conditions [42,43,44]. Advanced methods, such as a response surface methodology based on the knowledge of the critical input and output process parameters of secondary drying offer promising solutions for optimizing secondary drying by setting stop criteria based on residual moisture and estimating the required drying time [45]. Stability assessments post-drying are critical to verifying LAB’s survival rate and functionality and evaluating parameters such as cell viability, metabolic activity, and resistance to environmental stressors. Successful completion of the second drying phase culminates in a dry, stable product that can be stored long-term and easily reconstituted for various food, pharmaceutical, or biotechnology applications, thus fulfilling the primary objective of FD to preserve LAB cultures effectively [46].

### 2.4. Advantages and Disadvantages of Freeze-Drying

FD of LAB offers several significant benefits, which enhance their application in various industries, particularly food, pharmaceuticals, and biotechnology [47]. One of the primary advantages is the extended shelf life that FD provides. By removing moisture, the metabolic activities of the bacteria are halted, thus preventing spoilage and maintaining viability over long periods without refrigeration. This stability makes storage, transportation, and handling more convenient and cost-effective [48]. FD preserves heat-sensitive components by removing moisture at low temperatures, and maintaining the product’s original structure, including shape, size, and biological activity, ensuring long-term stability without degradation [49,50]. Additionally, FD retains the functional properties of LAB, such as their probiotic effects and fermentative abilities, ensuring their effectiveness upon rehydration. The process also allows for highly concentrated bacterial preparations, which are easier to dose and incorporate into products. Importantly, when used as probiotics, preserving cell integrity and bioactivity is essential to supporting gut health and immune function [51]. Though energy-intensive and costly, FD is critical in pharmaceuticals, food preservation, and historical document restoration, due to its effectiveness in maintaining the stability of sensitive materials [52,53]. Overall, freeze-drying enhances the practicality and efficiency of using LAB and maintains their beneficial properties, making them more versatile and reliable for various applications.

Despite its many advantages, FD LAB has several disadvantages that can impact their viability and functionality. One of the primary concerns is the potential for cellular damage during the FD process [54]. The formation of ice crystals during freezing can result in the piercing of cell membranes and the disruption of cellular structures, ultimately compromising the overall viability of the bacteria. Additionally, the process can cause oxidative stress and denaturation of proteins, which can impair the metabolic functions of the bacteria. Another significant drawback is the high cost of FD, which involves specialized equipment and energy-intensive processes [55]. Furthermore, the rehydration process must be carefully controlled, as improper rehydration can further lose viability and functionality [56]. These challenges highlight the need for the careful optimization and consideration of alternative preservation methods to ensure that the benefits of LAB are fully realized without compromising their quality and effectiveness.

## 3. Overview of Glycometabolism in Lactic Acid Bacteria

Glycometabolism in LAB involves the uptake, breakdown, and utilization of carbohydrates to produce energy in the form of ATP and lactic acid. The primary pathways facilitating this process are the Embden–Meyerhof–Parnas (EMP) and phosphoketolase (PK) pathways [21]. Additionally, LAB engages in the biosynthesis of exopolysaccharides (EPSs), further contributing to their metabolic activities.

### 3.1. Embden–Meyerhof–Parnas Pathway

**Figure 2:**
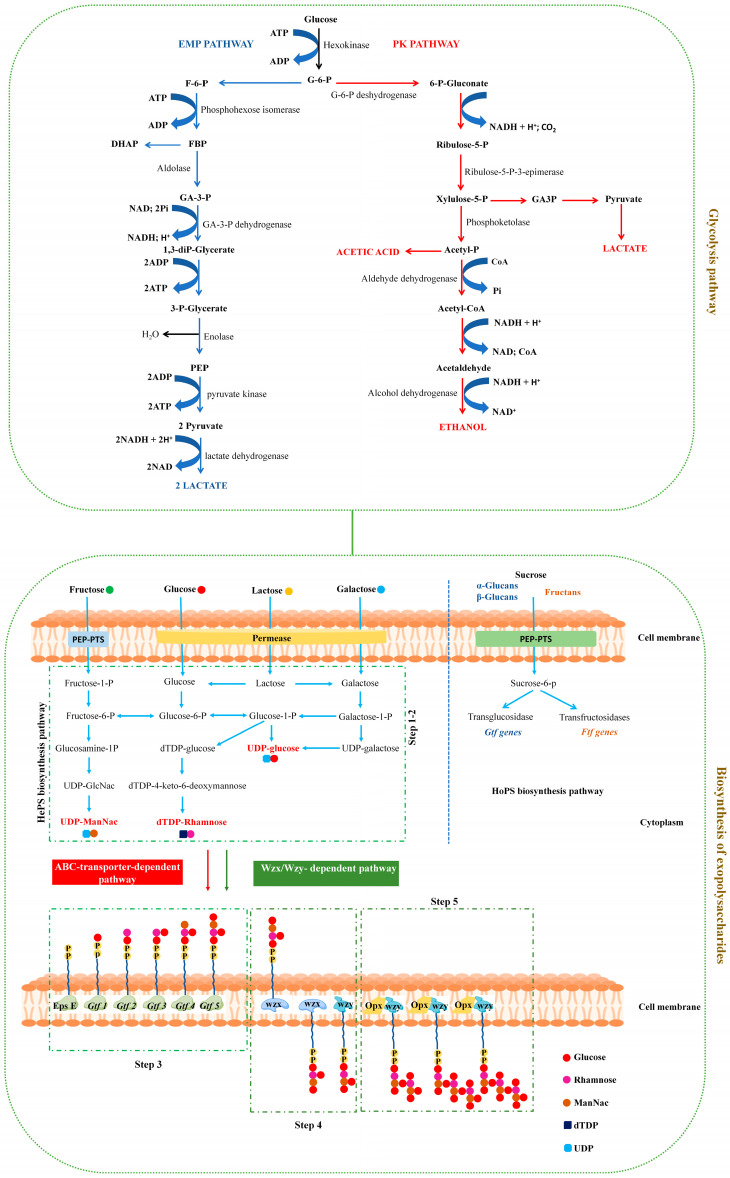
Two pathways of glycometabolism in the LAB during freeze-drying: Glycolysis pathway and exopolysaccharide biosynthesis pathway. Glycolysis pathway involves Embden-Meyerhof-Parnas pathway and phosphoketolase pathway, while exopolysaccharides biosynthesis involves both homopolysaccharide and heteropolysaccharide pathways. Latter includes five steps: (Step 1) transportation and phosphorylation of sugars; (Step 2) biosynthesis of sugars; (Step 3) synthesis of repeating units; (Step 4) translocation of sugars; and (Step 5) polymerization of repeating units and release of long chains. ADP: adenosine 5’-diphosphate; ATP: adenosine 5’-triphosphate; P: phosphate; NAD^+^: nicotinamide adenine dinucleotide; NADH: nicotinamide adenine dinucleotide (reduced form); Pi: inorganic phosphate; CoA: acetyl-coenzyme A; Wzx: flippase; Wzy: polymerase; Heps: heteropolysaccharides; Hops: homopolysaccharides; UDP: uridine diphosphate; dTDP: thymidine diphosphate; UDP-ManNac: UDP-N-acetylmannosamine.

The EMP pathway, also known as glycolysis, converts glucose into pyruvate, generating ATP and nicotinamide adenine dinucleotide (NADH), which are crucial for energy and redox balance in LAB [57]. There are several steps that the EMP pathway in LAB goes through (Figure 2): (i) glucose uptake; (ii) glucose phosphorylation; (iii) glycolysis; (iv) ATP and NADH generation; (v) lactic acid production; and (vi) energy generation. *Zymomonas mobilis* efficiently produces ethanol via the Entner–Doudoroff (ED) pathway but has a low biomass yield due to low ATP production. The ATP-rich EMP pathway stopped growth and caused glycerol production because of metabolic limits [58]. Glycolysis is pivotal for LAB survival, aiding in pH homeostasis and flavor compound formation in fermented foods and impacting sensory qualities [59]. Regulation mechanisms ensure efficient glucose use, adapting to environmental conditions. Additionally, EMP pathway intermediates contribute to biomass formation, which is essential for cellular growth and division. This pathway’s multifaceted role underscores its significance in LAB metabolism and industrial processes [60].

### 3.2. Phosphoketolase Pathway

LAB can metabolize pentose sugars such as xylose via the PK pathway, bypassing glycolysis. This metabolic route leads to lactic acid and ethanol production, thereby expanding the range of substrates that LAB can utilize for growth and fermentation [61]. Unlike glycolysis, the PK pathway involves distinct enzymatic reactions, beginning with the uptake of pentose sugars and their conversion to xylulose-5-phosphate (Figure 2). Phosphoketolase cleaves xylulose-5-phosphate into glyceraldehyde-3-phosphate (GAP) and acetyl phosphate (AcP), producing ATP. GAP continues through glycolysis to generate pyruvate, which is converted to lactic acid via lactate fermentation, while AcP forms acetyl-CoA, essential for fatty acid synthesis [62]. Certain *Lactobacillus* species utilize both the EMP and PK pathways simultaneously in the presence of glucose and xylose. These insights into carbon flow enhance the selection of strains and biomass for optimized lactic acid biosynthesis processes [63]. LAB thriving in pentose-rich environments efficiently utilize the PK pathway, demonstrating their metabolic adaptability across species. The intricate details of this pathway underscore LAB’s ecological versatility and metabolic diversity [64].

### 3.3. Exopolysaccharide Biosynthesis

EPSs are essential carbohydrates produced by microorganisms, including LAB, and play key roles in biofilm formation, tissue adhesion, and protection against environmental stress [65]. EPSs can be either homopolysaccharides (HoPSs) or heteropolysaccharides (HePSs), synthesized via distinct pathways, with HePSs primarily synthesized through the Wzx/Wzy-dependent pathway [66]. This process involves the Wzx flippase, which moves repeating sugar units to the outer membrane, where the Wzy polymerase extends the chain, regulated by chain length determinant protein (Wzz) or polysaccharide chain length regulator (PCP) [67]. The resulting EPSs help LAB survive in various environments by forming protective biofilms. The EPS gene cluster controls this pathway’s transport, polymerization, and chain length. Overexpressing epsC and Wzx increases EPS yield, adhesion, and resistance to low temperatures [68]. Overexpressing GalT in *L*. *acidophilus* increases EPS production and FD resilience by altering sugar utilization [69]. The synthesis of EPSs involves five steps: (i) sugar transportation and phosphorylation; (ii) the biosynthesis of sugar nucleotides; (iii) the synthesis of repeating units; (iv) the translocation of sugars and polymerization; and (v) the release of EPS chains (depicted in Figure 2).

## 4. Glycometabolism Modifications of Lactic Acid Bacteria Induced by Freeze-Drying

**Figure 3:**
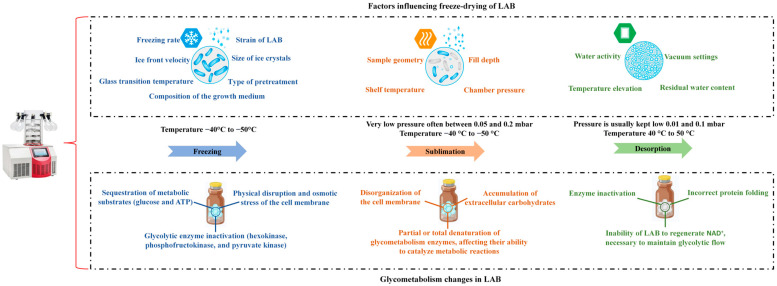
Glycometabolism changes and influencing factors in LAB during freeze-drying.

Various changes happen in the glycometabolic pathways of LAB when they are freeze-dried (Figure 3). These changes impact the reduction in glycolytic flux, oxidative stress response imbalance, and exopolysaccharide modifications. These alterations or changes are crucial for the survival and functionality of LAB after FD [18].

### 4.1. Reduction in Glycolytic Flux

Glycolysis plays a fundamental role in the metabolism of LAB, facilitating the conversion of glucose into pyruvate, which results in the generation of ATP and a reduction in NADH. This pathway is essential for energy production, particularly when oxygen is scarce. A variety of enzymes, such as hexokinase, phosphofructokinase, and pyruvate kinase, control the metabolic flow within glycolysis [70,71]. The activities of these enzymes are subject to modulation by environmental variables, including temperature, stress conditions, and nutrient availability. During FD, LAB undergoes severe stress due to rapid dehydration and exposure to low temperatures. These stressors significantly impact enzymatic activity, with ice crystal formation being a key factor in damaging the bacterial cell membrane. This disruption weakens the functionality of sugar transport systems, notably the Phosphoenolpyruvate-Phosphotransferase System (PEP-PTS), which hinders the uptake of glucose and other carbohydrates, thus limiting the substrates available for glycolysis [72,73]. Some studies suggest that the FD process could lead to substantial degradation of membrane integrity, but the presence of C18:1 may help maintain membrane fluidity and stability [74]. The reduction in glycolytic flux during FD is largely attributed to the deactivation of enzymes and the disruption of the bacterial cellular environment [13].

The FD process removes most of the water from the cells, which is critical for enzyme functionality and the solubility of substrates. The absence of water results in reduced mobility of enzymes and substrates, which consequently diminishes the efficiency of glycolytic reactions. Enzymes like hexokinase and pyruvate kinase are especially vulnerable to dehydration, showing decreased activity in the absence of sufficient water [75]. Moreover, the freezing step in FD leads to ice crystal formation, which can cause protein denaturation and damage to enzyme structures. This disruption is particularly problematic for glycolytic enzymes, as their proper functioning is vital for efficient glucose processing. As a result, the enzymatic damage impedes glycolytic flux after rehydration, which undermines the cell’s ability to regenerate ATP and maintain its essential functions [26]. Overall, the combined effects of reduced glycolytic flux during FD severely impact LAB’s energy production, viability, fermentation capacity, and their ability to function effectively after rehydration [76].

### 4.2. Oxidative Stress Response Imbalance

FD induces an oxidative stress response in LAB, primarily marked by an imbalance in the NADH/NAD^+^ ratio. As water is removed and cells are exposed to oxygen, reactive oxygen species (ROS) are generated, which can oxidize and inactivate key enzymes involved in glycometabolism [77]. Consequently, this oxidative stress disrupts the redox balance within the cell, impairing metabolic processes that depend on NAD^+^ and NADH. For example, lactate dehydrogenase, which converts pyruvate to lactic acid, requires a balanced NADH/NAD^+^ ratio to function efficiently. NADH accumulates when this balance is disturbed, leading to glycolytic inhibition and interference with the electron transport chain [78]. As a result, ATP production declines, diminishing cellular energy reserves and further threatening LAB’s viability and metabolic activity during FD.

In addition to affecting energy production, oxidative stress can damage genetic material, altering the expression of genes involved in carbohydrate metabolism and intensifying the metabolic shifts triggered by FD [79]. Notably, extensive research on oxidative stress responses was conducted across diverse LAB strains, including starter cultures (*Lactococcus lactis*), probiotics (various *Lactobacillus* species), and even pathogenic bacteria (*Enterococcus* and *Streptococcus* species). Given these impacts, addressing oxidative stress and NADH/NAD^+^ imbalance is essential for preserving LAB viability and functionality [80]. A study showed that *Oenococcus oeni* with FD and FD with monosodium glutamate (MSG) showed severe cell damage in the cells with FD and less membrane transmissibility in FD with MSG cells. Bioinformatic analysis revealed that varying proteins involved carbon, stress response, and oxidoreductase activity [81]. Therefore, ongoing studies focusing on stress mitigation strategies are critical for improving LAB resilience, enabling their effective application in industrial and probiotic formulations.

### 4.3. Exopolysaccharide Modifications

Survey results indicate a link between the composition, molecular weight, and yield of EPSs and their impact on the FD protective effect. (i) Composition and structure: Certain EPS varieties with robust FD resistance share similarities in composition and structure, notably rich in mannose [82]. For instance, *O. oeni*, a lactic acid bacterium used in winemaking, produces EPSs with the highest mannose content at 43%. Adding 2.5% of this bacterium’s EPSs increases FD survival rate to 81.43%, which is higher than common preservatives like 10% sucrose, 10% trehalose, and a mix of FD protectants. (ii) Molecular weight: EPSs with a high molecular weight exhibit increased heat absorption and strong water-holding capacity, mitigating cell damage from water loss during FD [83]. A 7.0 × 10^6^ Da molecular weight glucan helps mycelium grow after freeze–thaw cycles and makes strains more resistant to freezing environments with low temperatures [84]. (iii) Yield: Elevated EPS production is a protective response for LAB adapting to environmental changes. EPSs envelop bacteria, safeguarding cell integrity and enhancing strain tolerance to FD stress [85].

Glucan adhering to *L. plantarum* surfaces increases EPS production, which reduces 65.8% of the harm bacterial biofilm causes [86]. A quantitative relationship exists between EPS production and survival rate. *L. acidophilus* survival rates rise with increased EPS production under low-temperature conditions [87]. However, heating *L. acidophilus* to 45 °C for 30 min before FD causes significant changes in the structure, yield, and survival rate of EPSs: the yield goes up by 47.6%, the glucose ratio goes down from 21% to 17%, and the galactose content goes up from 17% to 26%. After FD, the process preserves cell wall and membrane integrity, increasing the survival rate from 39.1% to 56.3% (*p* < 0.05) [13]. The Wzx/Wzy-dependent EPS synthesis pathway affects the make-up, structure, yield, and other features of the EPS that is produced by creating different sugar nucleotides, managing the creation, movement, and polymerization of repeating units, and being a crucial part of LAB’s ability to withstand FD stress.

## 5. Factors Influencing Glycometabolism in Lactic Acid Bacteria

Several factors can affect the glycometabolism of LAB during FD (Figure 3). Since LAB are commonly used in food fermentation and probiotics, understanding how their glycometabolism is affected during FD is important for maintaining their viability and functionality.

### 5.1. Before Freeze-Drying

#### 5.1.1. Strain-Specific Differences

Recent studies have shown that the particular LAB strain affects the glycometabolism of LAB during FD (Table 1). Variations in the ability of LAB strains to metabolize carbohydrates can impact their survival and viability during FD [88]. Selecting LAB strains significantly affects glycometabolism, with some strains demonstrating a heightened capacity to synthesize glycogen. This glucose storage form aids in bacterial protection during FD. Conversely, other strains may exhibit a greater ability to utilize trehalose, a sugar that contributes to bacterial stabilization during FD [87]. Researchers have found that in FD, certain strains of *Streptococcus thermophilus* can change their glycometabolism, especially the levels of different sugars and sugar derivatives. This change could affect the bacteria’s performance in fermented dairy products [89].

Moreover, certain LAB strains display increased resistance to osmotic stress, enhancing their ability to maintain glycometabolism during FD. Because of this, some LAB strains are stronger and better able to handle the stresses of FD, while others may be more vulnerable, which could cause a drop in glycometabolism [90]. For example, different strains of *L. bulgaricus* have different enzyme activity levels in glycometabolism, which can cause differences in how well they survive FD [91].

#### 5.1.2. Pretreatment Techniques

The pretreatment of LAB before FD can significantly impact the glycometabolism of the bacteria. According to a study, the type of pretreatment, including whether it was thermal, osmotic, or acidic, significantly impacted the glycometabolism of LAB during FD [92]. Subjecting LAB cells to thermal stress, like heat shock or high temperatures, before treatment increased the activity of glycolytic enzymes and the levels of ATP inside the cells. These are important for cell survival and metabolic activity [13]. On the other hand, osmotic stress before treatment, like being exposed to high salt levels or dehydration, increases glycolytic enzyme activity and the amount of ATP inside the cells [93]. This suggests that osmotic pretreatment may better preserve the glycometabolism of LAB during FD. Additionally, exposing cells to low pH conditions before acid stress treatment increased the activity of glycolytic enzymes and the amount of ATP inside the cells. However, prolonged exposure to low pH conditions can be detrimental to the viability of LAB. In conclusion, the type of pretreatment used for LAB before FD can significantly impact the glycometabolism of the bacteria.

#### 5.1.3. Growth Medium Components

The growth medium used to cultivate LAB before FD can significantly impact their glycometabolism (Table 2). The composition of the growth medium can affect the concentration and type of carbohydrates present in the bacterial cells, which can influence their ability to survive and function after FD [94]. For example, a growth medium with high glucose levels can accumulate intracellular trehalose, which protects bacterial cells against various stresses, including FD. On the other hand, a growth medium with lactose may cause intracellular galactose to build up, making LAB more viable during FD [95]. Another study discovered that using a growth medium with fructose as the only carbon source significantly increased the production of EPSs. These complex sugars can protect cells even more during FD [96]. In addition to the carbon source, the composition of the growth medium can also affect the levels of other nutrients and metabolites that can impact the glycometabolism of LAB during FD. For example, the concentration of nitrogen sources, such as amino acids and peptides, can affect the production of EPSs and other carbohydrates. Minerals like calcium and magnesium can also change the glycometabolism of LAB by changing the activity of enzymes that break down carbohydrates [97].

### 5.2. During and After Freeze-Drying

#### 5.2.1. Freeze-Drying Parameters

FD time and temperature can significantly impact LAB’s glycometabolism. In LAB, glycometabolism is vital for producing organic acids, which play a role in fermented foods, flavor, texture, and preservation [27]. However, studies have shown that the time and temperature of FD can impact the glycometabolism of LAB in several ways. For example, longer FD times and lower temperatures have increased intracellular trehalose levels. This sugar is known to protect cells from environmental stresses. However, these conditions can also lead to decreased levels of other sugars, such as glucose and fructose, which can impact the ability of the cells to grow and metabolize [102]. For example, a study on *L. plantarum* found that increasing the FD temperature from −40 °C to −20 °C led to a decrease in the activity of enzymes involved in glycolysis, which is the breakdown of glucose to produce energy.

Additionally, increasing the FD time from 24 h to 48 h led to decreased enzyme activity in the pentose phosphate pathway, another pathway for glucose breakdown [30]. Conversely, shorter FD times and higher temperatures can lead to decreased levels of trehalose but increased levels of other sugars, such as glucose and fructose. This can improve the cells’ growth and metabolic activity following rehydration [99]. For example, one study looked at the impact of different drying temperatures (20 °C, 40 °C, and 60 °C) and drying times (24, 48, and 72 h) on the glycometabolism of *L. plantarum*. The results showed that higher temperatures and longer drying times decreased the activity of enzymes involved in glycolysis and the pentose phosphate pathway, which are essential for LAB’s ability to process sugars [22].

The pH level greatly affects glycometabolism in LAB during FD. It does this by changing the stability of enzymes, the shape of proteins, the transport of substrates, the production of metabolites, and the stability of cofactors. Maintaining an optimal pH throughout the FD process is crucial to minimizing these impacts. Cryoprotectants and buffer systems can help stabilize pH and preserve the metabolic activity of LAB [26]. The ability of LAB, especially *L. plantarum* LIP-1, to grow and survive in harsh conditions like FD depends on the initial pH of the medium. This study explored how initial pH impacts FD survival rates, cell membrane fatty acid composition, and critical enzyme activity. Results showed that an initial pH of 6.8 improved survival rates due to the upregulation of lactate dehydrogenase, leading to a rapid culture pH decrease. This stimulation upregulated the synthesis of genes and proteins, enhancing cell membrane integrity [23].

#### 5.2.2. Water Activity

Water activity (aw) is a critical determinant of microbial metabolism, especially during FD, as it regulates the availability of free water necessary for biochemical reactions that have an important influence on viability [103]. When the aw content of the powdered products is below 0.3, the biochemical reactions will decrease and have a positive effect on the shelf life of the products [104]. For freeze-dried bacteria, water availability is essential for enzymatic reactions, nutrient transport, and metabolite synthesis. Maintaining low aw during storage enhances bacterial viability [27]. The FD process removes intracellular water from bacteria until a low water activity level (aw ≤ 0.2) is reached, leading to a reduction or cessation of cellular metabolic activities [105].

The viability of freeze-dried LAB is influenced by various factors, including drying temperature, storage conditions, and water activity. Carbohydrates protect bacterial membranes through hydrogen bonding, preventing phase transitions during dehydration and protein denaturation. High carbohydrate concentrations form an amorphous glassy matrix that inhibits harmful reactions, and LAB stored below the glass transition temperature (Tg) in this matrix are better protected. Recent studies have highlighted the importance of storage temperature, water activity, Tg, and bacterial viability in preserving LAB during FD and storage [14]. As water activity decreases, bacteria enter a more dormant state, slowing down metabolic activities crucial for survival, which prevents the degradation of bacterial cells and their premature death under. Excessive aw > 0.3 can cause strong oxidation, which affects sample storage and loss of viability [106]. Water activity in freeze-dried *L. plantarum* ranged from 0.515 to 0.882. Despite the high aw, the cultures maintained viability, with a rapid decline in survival during the first 30 days, which then slowed due to the effects of cryoprotectants [107].

## 6. Strategies to Mitigate Freeze-Drying Effects

**Figure 4:**
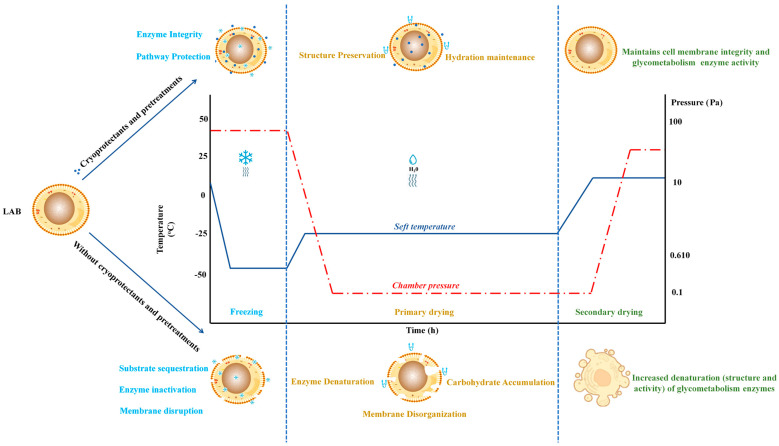
Benefit of cryoprotectants on LAB cell integrity and glycolytic enzyme activity during freeze-drying.

FD, while essential for the long-term preservation of LAB, imposes significant stresses that can disrupt glycometabolism and compromise cellular functions. Various strategies were developed to counteract these adverse effects and enhance LAB’s survival and metabolic functionality during and after FD [108]. For this reason, strategies such as cryoprotectants, lyoprotectants, encapsulation, and genetic or metabolic engineering are employed to preserve membrane integrity and protect LAB metabolic pathways during and after FD (Figure 4).

### 6.1. Cryoprotectants and Lyoprotectants

Cryoprotectants and lyoprotectants play a pivotal role in safeguarding the glycometabolism of LAB during FD by minimizing cellular damage and preserving metabolic activity [24] (Figure 4). Cryoprotectants, such as sucrose, trehalose, sorbitol, glycerol, and maltodextrin, function primarily by forming a protective glassy matrix around cells, immobilizing water molecules and reducing ice crystal formation. This vitrification process maintains the structural integrity of cell membranes and stabilizes key metabolic enzymes, including hexokinase, PFK, and pyruvate kinase, which are essential for sustaining glycolytic flux. [109]. A study indicated that the FD survival rate of *Lactococcus lactis* ZFM559 reached 81.02 ± 0.32% with a cryoprotectant mixture of 4.2% trehalose, 2.0% mannitol, 11.9% skim milk, and 4.1% monosodium glutamate. This combination preserved cell shape and provided external protection. Additionally, it increased the glass transition temperature, reducing ice crystal damage while maintaining cell membrane integrity and Na^+^/K^+^-ATPase activity, supporting metabolic stability [110]. Among these cryoprotectants, trehalose has received significant attention due to its ability to preserve the three-dimensional structure of proteins and enzymes by replacing water molecules and acting as a stabilizing agent during dehydration [111]. Furthermore, trehalose is an alternative energy source during post-FD recovery, facilitating metabolic reactivation and improving LAB viability (Table 3).

Lyoprotectants, on the other hand, are specifically designed to counteract stresses encountered during the desiccation phase of FD [125]. Compounds such as skim milk powder, whey proteins, and hydrocolloids form a physical shield around cells, preventing mechanical damage and stabilizing intracellular components. In addition, amino acids like proline and glutamate, often used as lyoprotectants, mitigate oxidative stress by acting as scavengers of ROS and maintaining redox homeostasis. Using a lyoprotectant composed of amino acids (glycine, arginine) and salts (NaHCO_3_ and ascorbic acid) (glycine 4.5%, arginine 5.5%, NaHCO_3_ 0.8%, and ascorbic acid 2.3%), under these optimal conditions, the survival rate of lyophilized powder of *Bifidobacterium bifidum* BB01 was significantly elevated to 80.9% compared with the control group (6.9 ± 0.62%) [126]. These protective effects preserve the NADH/NAD^+^ balance, which is crucial for properly functioning glycolytic enzymes and energy metabolism. The combined use of cryoprotectants and lyoprotectants enhances LAB’s survival and metabolic stability during FD. By stabilizing cell membranes, protecting key enzymes, and mitigating oxidative stress, these compounds ensure the maintenance of glycolytic flux and overall cellular functionality [79]. Optimizing the formulation and concentration of cryoprotectants and lyoprotectants is essential for achieving the best protective effects. For instance, a combination of trehalose as a cryoprotectant and skim milk as a lyoprotectant has significantly improved LAB’s survival rates during FD.

### 6.2. Encapsulation

Encapsulation has emerged as a robust strategy to mitigate the deleterious effects of FD on LAB. During FD, LAB are subjected to extreme conditions, including low temperatures, vacuum, and dehydration, which can significantly impair their viability and functionality [127]. To address these challenges, encapsulation involves embedding LAB within protective materials, such as polysaccharides, proteins, or lipids, thereby creating a shielded microenvironment. This protective layer safeguards the bacteria from direct exposure to harsh conditions and enhances their stability during storage [128]. For instance, *L. acidophilus* KBL409 encapsulated in alginate/chitosan (Al/Chi-) *L. acidophilus* KBL409 with or without sucrose were prepared and evaluated for simulated gastrointestinal tract (GI tract), moisture characteristics, and storage stability. Al/Chi cells freeze-dried with sucrose showed the highest survival rate of 0.14% in the GI tract (*p* < 0.05) [129]. It was shown that the encapsulation of *L. casei* ATCC 393 cells with pea protein isolate–alginate hydrogel matrix after FD achieved a high yield of 85.69% ± 4.82, indicating that the matrix and encapsulation technique were compatible with the probiotic strain. In addition, the dried capsules were subjected to further storage tests at three temperatures (+22, +4, and −15 °C). After 84 days of storage, the encapsulated *L.casei* stored at −15 °C showed the highest survival rate among all samples (59.9% ± 17.4) [122].

Moreover, the controlled release of encapsulated LAB ensures they are delivered effectively to target sites, which is particularly advantageous for probiotic applications. Additionally, the encapsulating material can act as an antioxidant, reducing oxidative stress on the bacteria during the FD process [127]. Therefore, incorporating encapsulation techniques into the FD protocol can substantially enhance LAB’s overall efficacy and longevity, making it a critical consideration for glycometabolic studies and practical applications (Table 3).

### 6.3. Genetic and Metabolic Engineering

Genetic and metabolic engineering strategies present sustainable solutions for bolstering the resilience of LAB glycometabolism during FD. Leveraging advancements in molecular biology, LAB can be genetically modified to overproduce protective biomolecules, such as trehalose-phosphate synthase, exopolysaccharide synthase, and stress-responsive proteins like heat shock proteins (HSPs) and cold-shock proteins (CSPs) [130]. Enhancing the expression of trehalose-phosphate synthase elevates the intracellular accumulation of trehalose, a recognized cryoprotectant that stabilizes glycolytic enzymes, including PFK and pyruvate kinase.

Metabolic engineering approaches aim to rewire the glycolytic pathway and boost the production of key protective metabolites. For instance, amplifying the expression of genes encoding PFK and pyruvate kinase enhances glycolytic flux, ensuring a consistent supply of pyruvate vital for cellular energy production and redox balance [131]. Diverting glucose metabolism toward synthesizing EPSs and trehalose offers additional protection, as EPSs form a physical barrier against ice crystals, and trehalose stabilizes glycolytic enzymes. Furthermore, genetic engineering can augment the antioxidant capacity of LAB by increasing the production of glutathione or catalase, which neutralizes ROS and safeguards glycolytic enzymes from oxidative damage.

## 7. Future Applications of Lactic Acid Bacteria with Modified Glycometabolism

It is important to note that any practical implementation of LAB with modified glycometabolism would require rigorous safety assessments and ethical considerations. Genetic modifications to microorganisms must be carefully evaluated for potential unintended consequences and environmental impacts. Nevertheless, the potential prospects and possibilities can be, among others, probiotics and gut health. LAB is commonly used as probiotics to improve gut health [132]. LAB might be better able to ferment certain carbohydrates if their glycometabolism is changed. This could increase the production of beneficial metabolites like short-chain fatty acids (SCFAs). SCFAs play essential roles in gut health, including maintaining gut barrier function and promoting immune regulation. Changing how LAB’s glycometabolism works could lead to probiotics with specific functions, which could help treat several gastrointestinal problems [133]. Next are prebiotics and synbiotics, which are non-digestible food components that promote the growth and activity of beneficial gut bacteria, including LAB. Scientists might be able to make new synbiotics by changing the glycometabolism of LAB to target specific prebiotics. Synbiotics are mixes of probiotics and prebiotics that work together to improve gut health. This approach could lead to more targeted and effective formulations to promote a balanced gut microbiota [94].

LAB is widely used in the food industry for fermentation processes, helping to produce a variety of fermented foods and beverages. LAB could better break down certain sugars in raw materials if their glycometabolism were changed [134]. This improvement would enhance fermentation efficiency and create new, healthier, or tastier products. Concerning bioproduction of biofuels and bioplastics, lactic acid, an essential product of LAB metabolism, is a precursor for biofuels and bioplastics. Researchers may improve LAB’s ability to make lactic acid by changing their glucose use, making them more efficient biofactories for producing these sustainable materials [135]. Engineered LAB with modified glycometabolism can be used as delivery vehicles for therapeutic compounds, vaccines, and gene therapies. Their ability to target specific tissues or cells could be improved, increasing the efficacy of treatments and reducing side effects.

## 8. Conclusions

LAB offers numerous advantages in various applications due to their beneficial properties. We usually use FD to preserve LAB because it can extend shelf life and maintain cell viability. While it offers several advantages, including long-term stability, accessible transport, and reconstitution, it also has some drawbacks. The FD process was shown to change the glycometabolism of LAB. These changes can be attributed to decreased glycolytic flux and an imbalance in the oxidative stress response, resulting from ice crystal formation and water loss that disrupt the cellular environment. This article talked about how the glycometabolism of LAB changes and how it works during FD, focusing on how the glycometabolism of LAB breaks down during FD. Moreover, it shows factors influencing the glycometabolism of LAB and applications to improve the impact of lyophilization on the mechanism of glycometabolism in LAB. Understanding and managing these changes is crucial for maintaining the functionality and viability of LAB cultures during freeze-drying, ensuring their effectiveness in various applications such as probiotics, fermented foods, and biotechnology. Further research is needed to explore strategies for optimizing glycometabolism during FD and improving the performance of rehydrated LAB cultures.

## Figures and Tables

**Table 1:** Stress conditions affecting LAB species.

Influencing Factors	Stress Condition	LAB’s Species	Results Observed	References
Heat shock	37 °C for 10 h	*Lactobacillus acidophilus* NCFM	GalU activity ↑, survival rate ↑.	[14]
	45 °C for 30 min	*L. acidophilus* ATCC4356	Survival rate ↑, glucose-6-phosphate isomerase, lactate dehydrogenase, phosphoglucomutase, UDP-glucose pyrophosphorylase, glycosyltransferases, Na^+^-K^+^ -ATPase ↑	[13]
	52 °C for 2 h	*L. kefiranofaciens* M1	Triosephosphate isomerase, enolase, and NAD-dependent glycerol-3-phosphate dehydrogenase ↑; chaperones DnaK and GroEL ↑	[15]
Cold	−5 °C	*Leuconostoc mesenteroides* WiKim32	Thickness of exopolysaccharides ↑ Viability of freeze-dried *Leu. mesenteroides* WiKim32 ↑	[16]
	−5 °C for 2 h	*L. brevis* WiKim0069	Thickness surface layer protein ↑ Cell viability ↑	[17]
Salt	2% (*w*/*v*) NaCl for 2 h	*L. bulgaricus*	Survival rate ↑ Glycolytic enzymes (phosphofructokinase, pyruvate kinase, and lactate dehydrogenase) ↑	[18]
Acid	pH 3.5 for 2 h	*Bifidobacterium longum* sub. *longum* BBMN68	Energy production, amino acid metabolism, and peptidoglycan synthesis during the ASR ↑ ATP, NH3, thiols; and peptidoglycan, the activity of H^+^-ATPase ↑	[19]
Starvation	Carbohydrate starvation	*L. casei* Zhang	Carbohydrate metabolic, lyase activity, and amino acid-transporting ATPase activity ↑	[20]

Abbrevitions: ASR, acid stress response; ATP, adenosine triphosphate; DnaK, heat shock protein; GroEL, chaperonin; H^+^-ATPase, hydrolysis of adenosine triphosphate; NAD, nicotinamide adenine dinucleotide; Na^+^-K^+^-ATPase, sodium–potassium adenosine triphosphate; UDP, uridine diphosphate; ↑, increases.

**Table 2:** Preservation and growth factors for LAB species.

Influencing Factors	Stress Condition	LAB’s Species	Results Observed	References
Growth medium	Trehalose and Lactose are often used as protective agents	*L. fermentum* FXJCJ6-1, *L. brevis* 173-1-2, *L. reuteri* CCFM1040	FD resistance ↑	[95]
	Glucose, trehalose, sucrose, and sorbitol	*L. plantarum*, *L. rhamnosus* GG	FD resistance ↑	[98]
Time and temperature	Frozen at −20, −80, and −196 °C	Yeasts: *Pichia membranifaciens*, *Starmerella bacillaris*, *Metschnikowia pulcherrima*; LAB: *L. paracasei*, *Pediococcus parvulus*, *L. mali*	Survival rates ↑ Starter production processes ↑	[99]
	Frozen at −80 °C	*Lactobacillus*, *Streptococcus*, *Lactococcus*, *Enterococcus*	Viability, autolytic activity, and intracellular enzymatic activity ↑	[100]
Storage	Stored at −20 °C	*Enterococcus faecium* MC13	Cell viability of bacteria containing trehalose ↑	[101]
	Stored at −20 °C	*Lactobacillus*, *Streptococcus*, *Lactococcus*, *Enterococcus*	After six months of storage, survival rate ↑ and intracellular enzymatic activity ↑	[100]

Representation: ↑, increases.

**Table 3:** Effects of different cryoprotectant/encapsulation and freeze-drying parameters on LAB viability.

Cryoprotectant/Encapsulation	Strain	Medium	Optimization of FD Process Parameters	Viability	References
4.2% trehalose, 2.0% mannitol, 11.9% skim milk, and 4.1% glutamic acid monosodium	*Lactococcus lactis* ZFM559	M17 broth	Frozen at −80 °C for 2 h, FD at 0.1 mPa for 30 h	81.02 ± 0.32%	[110]
8.2% sucrose, 8.0% skim milk, and 8.2% inulin	*L. plantarum* grx16	MRS	Frozen at −60 °C for 12 h, FD at −50 °C, 0.4 Pa for 24 h	94.9%, 1.89 × 10^11^ cfu/g	[112]
Ectoine, trehalose, and sucrose	*Streptococcus thermophilus*	M17 broth	FD and subsequent storage at −80 °C	Ectoine: 204 ± 2.46% Trehalose: 174 ± 3.73% Sucrose: 117 ± 1.44%	[113]
			FD and subsequent storage at −20 °C	Ectoine: 181 ± 0.78% Trehalose: 156 ± 3.17% Sucrose: 103 ± 1.22%	
			FD and subsequent storage at +4 °C	Ectoine: 107 ± 3.21% Trehalose: 93 ± 2.26% Sucrose: 61 ± 1.91%	
1% L-theanine	*L. plantarum* MG5023, *Enterococcus faecium* MG5232, *Lactococcus lactis* MG4668, *Streptococcus thermophilus* MG5140, *Bifdobacterium Animalis* subsp. *lactis* MG741	MRS broth	Frozen at −80 °C, FD at −30 °C, 0.5 mbar for 24 h	MG5023: 96.97%, MG5232: 75.80%, MG4668: 64.21%, MG5140: 76.41%, MG741: 65.29%	[114]
11.1% trehalose, 9.1% glycerin, 3.5% sodium glutamate, and 15.7% skimmed milk powder	*L. rhamnosus*	MRS	Frozen at −80 °C for 48 h using a vacuum freeze-dryer ALPHA 2–4 LD plus	97.8%	[40]
6% sucrose/ 8% skim milk/ 4% sodium glutamate	*Streptococcus thermophilus*	M17	Pre-freeze at −60 °C for 12 h, FD at −50 °C, 0.4 Pa for 24 h	90.59% and 1.78 × 10^11^ CFU/g	[115]
Skim milk, 10% trehalose, 10% lactose, 10% sucrose, glycerin, sorbitol, and Tween-80	*L. plantarum* L1 and *L. fermentum* L2	MRS broth	Pre-frozen at −80 °C for 4 h, FD at −55 °C, 0.06 mbar for 24 h	The survival rates of L1 and L2 in 10% Skim milk was 37.4% and 46.6%	[116]
Skim milk, inulin, maltodextrin, and sucrose	*L. plantarum*	MRS	Pre-frozen at −80 °C for 24 h, FD −35 °C for 24 h.	Skimmed milk demonstrated the highest survival rate, at 91%	[117]
1% soy polysaccharides, 10% mannitol, 10% sucrose, and 10% trehalose.	*L. plantarum* Strains AR113 and AR307, *L. plantarum* WCFS1	MRS broth	Pre-frozen at −40 °C for 3 h, FD at −30 °C, 20 Pa for 13 h, 25 °C for 20 h	WCFS1’s survival rates increased 2.9-fold (40.93%) with trehalose and 5.5-fold (77.84%) with sucrose compared to the control (14.14%), while AR307’s survival rate was 13.5 times higher (94.3%) with trehalose	[97]
Sodium alginate and pumpkin powder	*L. plantarum* MG989, *L. fermentum MG901*, *Streptococcus thermophilus* MG5140, *L. lactis* MG5125, and *Enterococcus faecium* MG89-2	MRS broth	Primary drying at −40 °C for 1 h, secondary drying up to 20 °C over 24 h.	Survival rate enhanced up to 28.7%	[118]
10% sucrose, 5% trehalose, 10% skim milk powder, and 10% skim milk powder + 5% sodium glutamate	*L. fermentum* IAL 4541 and *Wickerhamomyces anomalus* IAL 4533	MRS broth	Pre-frozen at −80 °C for 12 h, FD at −50 °C, 0.15 mbar for 24 h.	87% of cell viability was observed after one year storage	[119]
5% sucrose contained 10% skim milk and 2% pumpkin powder	*L. plantarum* MG989, *L. fermentum* MG901, *Streptococcus thermophilus* MG5139, *L. lactis* MG534, *Enterococcus faecium* MG89-2, and *Bifidobacterium animalis* ssp. *lactis* MG741	MRS	Not available	43.1 to 86.6% cell viability was observed during the FD process	[120]
Trehalose	*Mammalian cells*	MRS	Not available	62 ± 20%	[15]
Protein isolate (WPI), protein isolate + inulin, protein isolate + inulin + Persian gum	*L. rhamnosus* ATCC 7469	MRS broth	Freezing at −80 °C, FD at 0.02 mbar for 24 h.	Viability of encapsulated cells decreased from 10.60 to 10.97 log cfu/g in the first week to 8.72–10.78 log cfu/g in the last week of storage	[121]
Protein isolate–alginate hydrogel	*L. casei* ATCC 393	MRS broth	Frozen −80 °C for 8 h, FD for 48 h	85.69% ± 4.82	[122]
Whey protein isolate, alginate	*L. plantarum* VAL6 and *L. acidophilus* VAR1	MRS	Frozen at −70 °C for 1 h FD at −50 °C, 0.6 mpa for 24 h	VAL6 ’s survival rate 43.61% VAR1’s survival rate 73.33%	[87]
Trehalose matrix	*L. rhamnosus* GG	MRS broth	Frozen at −60 °C for 3 h FD at − 40 °C, 4 Pa for 72 h	49.58%	[123]
Skim milk, sucrose, maltodextrin, and corn starch	*L. acidophilus* FTDC 3081	MRS broth	FD at − 51 °C for 24 h	80%	[124]

## Data Availability

No new data were created or analyzed in this study. Data sharing is not applicable to this article.
